# Medicine and management in European hospitals: a comparative overview

**DOI:** 10.1186/s12913-016-1388-4

**Published:** 2016-05-24

**Authors:** Ian Kirkpatrick, Ellen Kuhlmann, Kathy Hartley, Mike Dent, Federico Lega

**Affiliations:** Leeds University Business School, University of Leeds, Leeds, LS29JT UK; Medical Management Centre, LIME, Karolinska Institutet Stockholm, Sweden, and Institute of Economics, Labour and Culture, Goethe-University Frankfurt, Frankfurt, Germany; University of Salford, Salford Business School, Lady Hale Building, Salford, M5 4WT UK; Staffordshire University, College Road, Stoke-on-Trent, Staffordshire ST4 2DE UK; Department of Policy Analysis and Public Management, Bocconi University, Via Safratti, 25, Milan, Italy

**Keywords:** Medicine, Management, Leadership, Public hospitals, Comparative research, Hybrid roles, Performance

## Abstract

**Background:**

Since the early 1980s all European countries have given priority to reforming the management of health services. A distinctive feature of these reforms has also been the drive to co-opt professionals themselves into the management of services, taking on full time or part time (hybrid) management or leadership roles. However, although these trends are well documented in the literature, our understanding of the nature and impact of reforms and how they are re-shaping the relationship between medicine and management remains limited. Most studies have tended to be nationally specific, located within a single discipline and focused primarily on describing new management practices. This article serves as an Introduction to a special issue of BMC Health Services Research which seeks to address these concerns. It builds on the work of a European Union funded COST Action (ISO903) which ran between 2009 and 2013, focusing specifically on the changing relationship between medicine and management in a European context.

**Main text:**

Prior to describing the contributions to the special issue, this Introduction sets the scene by exploring *four* main questions which have characterised much of the recent literature on medicine and management. First is the question of what we understand by the changing relationship between medicine and management and in particular which this means for the emergence of so called ‘hybrid’ clinical leader roles? A second question concerns the forces that have driven change, in particular those relating to the wider project of management reforms. Third, we raise questions of how medical professionals have responded to these changes and what factors have shaped their responses. Lastly we consider what some of the outcomes of greater medical involvement in management and leadership might be, both in terms of intended and unintended outcomes.

**Conclusions:**

The paper concludes by summarising the contributions to the special issue and highlighting the need to extend research in this area by focusing more on comparative dimensions of change. It is argued that future research would also benefit theoretically by drawing together insights from health policy and management literatures.

## Background

Since the early 1980s all European countries have given priority to reforming the management of health services. This trend is heavily influenced by ideas of the new public management (NPM) and the wider goal of increasing convergence between the practices of public organisations and private firms [1]. The result has been a marked change in the organisational and funding landscape of public hospitals, increasing their autonomy to make decisions locally, while, at the same time, intensifying demands to meet performance targets. In many respects these changes have posed a direct challenge to the dominance of clinical professionals (especially doctors) in the running of public hospitals. These professions exercise a key discretionary role in decisions about patient treatment that inevitably affect overall resource allocation, which governments and other payers have been keen to curtail or direct. However, a distinctive feature of these reforms has also been the drive to co-opt professionals themselves into the management of services [2]. This has involved doctors and nurses becoming full time managers, or part time ‘hybrid’ professional managers, such as clinical director roles or general practitioners with responsibility for budgets. More recently there have been calls to encourage a wider constituency of clinical professionals, including those who may never become managers, to engage in ‘leadership’ activities, willing and able to lead a reform of health services [3]. Indeed, with many professional bodies now actively supporting or – in some instances – driving these changes it would seem that clinical leadership has moved from ‘the dark side to centre stage’ [4].

These trends have been well documented in the literature. However, our understanding of the nature and impact of reforms and how they are re-shaping the relationship between medicine and management remains limited in a number of key respects. *First*, much of this work has a very strong national focus and with some exceptions has not explored comparative developments in the role that doctors (and other clinicians) are playing in management and leadership. *Second*, existing research is fragmented theoretically with studies addressing the issue of changing medical management roles from a variety of disciplinary standpoints, including: management, economics, social policy, sociology and medicine. *Third* are certain methodological limitations. Most researchers have relied either on case-study based methods or surveys, seeking to generalise across a wider population of organisations - with few attempts to combine the two or explore changes over time. *Finally*, most attention has focused on describing new management practices and the response of professionals to them, with (relatively speaking) less attention given to the impact and consequences of this change.

In this volume our aim is to begin to address some of these concerns. The papers that follow explore the changing relationship between management and medicine from a number of perspectives, focusing on different European health systems. Much of this work arises from a European Union funded COST Action (ISO903) which ran between 2009 and 2013. This Action, for the first time, helped to establish a network of academics and scholars from different disciplinary backgrounds focusing on developments in health management in a distinctly *European* context. The results of this work have been numerous, helping to deepen understanding of the institutional factors that have shaped reform (and reform outcomes) in different countries, and also extending empirical knowledge in key areas, such as governance, clinical leadership and comparative developments in policy and practice.

In what follows we provide an overview of this work, focusing on each of the contributions to the Special Issue in turn. However, prior to that we first set the scene by exploring *four* main questions which have characterised much of the recent literature on medicine and management. First is the question of what we understand by the changing relationship between medicine and management, in particular what this means for the emergence of so called ‘hybrid’ clinical leader roles? A second question concerns the forces that have driven change, in particular those relating to the wider project of NPM reforms. Third, we raise questions of how medical professionals have responded to these changes and what factors have shaped their responses. Lastly we consider what some of the outcomes of greater medical involvement in management and leadership might be, both in terms of intended and unintended outcomes.

### Terms of reference

As suggested above, our main point of departure for this volume is the understanding that the relationship between management and medicine is changing. On the one hand this can be understood as a process in which the work of clinical professionals (including doctors) is being increasingly managed by external parties. This might take the form of more intrusive forms of regulation, for example, defining forms of treatment that doctors may prescribe, interventions to alter medical training and competencies or rules governing clinical audit and reporting. These changes, which many argue, have slowly undermined the dominance and institutional autonomy of medical professionals are widely documented in the literature [2]. At the organisational level, the external management of medicine has also become more pronounced. This is evidenced by the changing employment status of doctors in some countries (limiting scope for private work), performance targets and tighter financial controls restricting clinical freedom.

However, while this narrative of encroaching bureaucracy should not be dismissed, at the organisational level it is important to raise questions about *who* is performing management? As noted earlier (and discussed below), a feature of health reforms has been the recruitment of new cadres of specialist managers who form a distinct occupation responsible for tasks of coordination and control, the allocation of resources (including staff) to meet performance objectives. More often than not these specialists will have no clinical background and may even come from the private sector with quite different skills and orientations [5]. This has been notably true in the UK which, following the Griffiths report (1983), pioneered the recruitment of general managers to run public hospitals [6]. Yet, while these managers have become part of the organisational landscape and have captured media attention (castigated as overpaid ‘men in grey suits’), they remain very small in number [7]. Recent estimates suggest that ‘management’ in the NHS (including central functions) accounts for less than 3 % of the workforce, compared with approximately 7 % of the UK workforce as a whole [7]. While reliable figures for other European health systems are hard to come by, the available evidence suggests that non clinical managers are, if anything, even fewer in number [8].

Given this it seems that a large amount of the actual day to day work of managing services will fall to doctors (and other clinicians) themselves. In some cases, clinical professionals will perform these duties without any formal recognition (as part of their day-to-day activity), while in others they may take on formal management roles, such as clinical directors or heads of department. In all cases one can note the emergence of so-called hybrid roles which occupy what Waring ([9], pp. 688–89) terms ‘liminal spaces’ where there is a “need to manage competing priorities, organisational inconsistencies and dual identities”.

This change may be having implications for the orientations and priorities of clinicians who become more involved in management. In the past, doctors who have turned to administrative work have tended to adopt ‘custodial’ orientations, focused on protecting existing collegial relations and practices [10]. Even today this response is quite common, as is noted in the papers by Correia and Denis, focusing on Portugal and Kuhlmann, Rangnitt and von Knorring looking at management reforms in Sweden. However, there are also a growing number of studies, which point to the growing willingness of some clinical managers to challenge professional practice [11]. Fitzgerald and Ferlie ([12], p.729), for instance, report how some clinical directors in the English NHS actively sought these positions, demonstrating a ‘crusading zeal for change’ by addressing ‘thorny’ issues such as differential levels of performance amongst colleagues’. In the Finish case Kurunmaki [13] goes even further, concluding that many doctors have embraced the techniques of accounting and, are no longer exclusively curative in their aspirations.

Another implication is that an increasing proportion of professional time is now devoted to ‘management’ or leadership activities. This is clearly greatest in situations where doctors take on formal roles such as clinical directors or sitting on the boards of hospitals. However, this may ignore a far larger group of what Causer and Exworthy [14] term ‘quasi managerial practitioners’, potentially including all doctors who – on a more ad hoc, informal basis - may take on occasional management roles, such as the supervision or mentoring of junior colleagues or non- professionals. By all accounts this may be quite extensive. In the English NHS for example, a recent study found that around one in three clinical staff had some kind of ‘managerial’ role [15]. The current emphasis on clinical leadership has also muddled the waters. This is especially so given the idea of internalising leadership roles, such as mentoring and innovation in service development, into the day to day work of doctors and other clinicians [3]. If anything this inclusive approach has been given a further boost by the notion of leadership as ‘collective’ or ‘distributed’ within teams of professionals engaged in change initiatives (a theme picked up in the paper by Denis and van Gestel, comparing Canada and the Netherlands).

### Drivers of medical involvement in management

As hinted at above, the increased management component in professional work has much to do with broader reforms of the sector, most notably those linked to the NPM [16]. Where health services are concerned it is worth highlighting two elements of these reforms, both of which have important implications for the changing relationship between medicine and management_._ First are wider structural changes that have altered the organisational landscape in which clinical professions work. Second are new organisational templates for how to manage hospitals (for a more extensive discussion also see the paper by Jeurissen, Duran and Saltman).

Starting with the organisational landscape, most public hospital systems in Europe have historically taken the form of vertically integrated hierarchies with tight controls over funding and staffing decisions exercised by national or regional tiers of government administration [17]. This meant that a great deal of the formal management (or administration) of services, such as those provided within hospitals, was performed at higher levels, out of sight and out of mind, from clinical professionals regulating their own practices within hospitals. Indeed, following Brunsson and Sahlin-Andersson ([18], p. 734) it is useful to think of hospitals as ‘incomplete organisations’, or better still, as ‘arenas’ ‘where members perform their tasks relatively free from control by the local leadership’.

NPM reforms have however, now started to transform this environment. Specifically, there have been moves to decentralise the management of services to more operational levels of health services and, at the same time, increase both the formal autonomy (and accountability for performance) of organisations such as hospitals [17]. This restructuring might involve the wholesale privatisation of public hospitals, although more likely it has involved ‘corporatisation’ – ‘a change in legal form that separates service delivery from traditional government agencies while keeping the organisation in public hands’ ([19], p. 2). Examples of this model can be found across European health systems, including ‘self-governing’ and ‘foundation trusts’ in England, limited liability companies in the Czech Republic, ‘public enterprise entity hospitals’ in Portugal, as well as a variety of different models in Spain [17].

Common to all of these changes has been the objective of transforming hospitals (or groups of hospitals) into more ‘complete organisations’ ‘by installing or reinforcing local identity, hierarchy and rationality’ ([18], p.721). Like firms in the commercial sector hospitals now have their own distinctive governance arrangements, made up of executive boards, with varying degrees of financial and operational autonomy. They also face increasing demands to improve performance and efficiency. In some cases this may originate from more intrusive forms of performance management and target setting – a fact which some observers claim, makes a mockery of the idea that hospitals are now independent. Growing external pressure is also linked to the way many countries now fund public hospitals, moving away from block incremental budgets to income streams that are more variable, linked to patient demand and the use of services (for instance diagnostic related groups). The latter is especially pronounced in those countries (such as the UK) where governments have tried to manufacture internal markets for services, formally separating the roles of commissioner and service provider.

Returning to our main concerns, these structural changes clearly have implications for the relationship between medicine and management. On the one hand, they may be viewed as a threat to the dominance of medicine, increasing the leverage of professional managers. However, at the same time, re-structuring may facilitate the growing involvement of doctors (and others) in the management process. This might be attributed to what Lindlbauer et al. [19] describe as the ‘symbolic effects’ of corporatisation and how it is presented. Radical changes in the funding and autonomy of hospitals can be regarded both as an external threat (to the very survival of the hospital) and as opportunity to innovate with new service design.

The second characteristic of NPM reforms worth noting here concerns the dissemination of alternative models for how to manage clinical services within hospitals. As hinted at above, historically, the default model of hospital organisation bore many of the hallmarks of a professional bureaucracy, with management formally separated from the ‘worlds’ of care and cure [20]. This usually involved parallel hierarchies, with doctors represented by a senior medical committee, not formally accountable to senior administrators or responsible (or even aware) of resource decisions. Administrators themselves tended to function as diplomats, focused on negotiating consensus between different stakeholders.

By contrast, NPM reforms have helped to usher in radically different models for how to internally organise (and manage) clinical operations. Perhaps the most influential of these is the model of clinical directorates (CDs), originally pioneered at the Johns Hopkins Hospital, a teaching hospital in Baltimore in 1972 but which has subsequently spread much more widely [21]. According to Braithwaite and Westbrook ([22], p. 142): ‘The clinical director (CD) concept dispersed relatively rapidly, in ways that innovation diffusion theorists would find predictable of an attractive idea’, such that ‘every large hospital now has some form of CD structure as a key component of its’ governance arrangements’. Other models have focused more on breaking down boundaries between clinical specialities and departments, encouraging inter-professional teamwork and collaboration around common processes or patient pathways [23] (see paper by Lega and Sartirana for a discussion of the Italian case).

As with the structural changes described above, these new organisational models are likely to intensify the pressure on medical professionals to engage in the management of services. This is most obviously true with regard to clinical directorates, which in most countries are associated with the development of ‘hybrid professional-manager’ roles, normally held by senior consultants. More ambitious proposals to develop service improvement networks and re-organise provision around patient pathways generate even more significant management challenges, requiring a wider constituency of clinical professionals to become involved in brokering and change leadership activities [24].

Hence, it is clear that global NPM reforms have been a critical driver of change in the relationship between medicine and management. However, while acknowledging this fact it would also be a mistake to ignore the agency of the medical profession itself in pushing for change. Indeed, one does not need to look far to uncover examples of this. In the UK the Royal College of Physicians ([25] p.xii) have redefined medical professionalism for the twenty first century as: ‘multiple commitments - to the patient, to fellow professionals, and to the institution or system within which healthcare is provided’. The General Medical Council also now requires doctors to be not only expert practitioners, but ‘partners,’ working with managers and other professionals, and ‘leaders’ of services [26]. Similar moves by professional bodies to engage with the changing educational requirements associated with management and leadership have been noted in other European countries, for example, Denmark and the Netherlands [27] (A theme picked up in the paper by Hartley).

### Professional responses and conditions shaping responses

So far we have described how medical professions in Europe now face intensified pressures to increase their involvement in management and leadership activities. However, it is far from clear to what extent this will translate into deeper levels of engagement and, when it does, how we might explain it. In this section we consider these questions, focusing on the likely obstacles to change and (briefly) on what we might learn from the existing research on ‘hybrid professional-manager’ roles. We also raise questions about the factors that might shape the response of medical professionals, including those relevant to the individual, organisational and national policy context.

Potential obstacles to deeper medical involvement in management and leadership are well documented in the literature. Hospitals have been described as places ‘where a number of tribes interact’, or, in the worst case, where managers and clinicians are locked into ‘oppositional stalemate’ ([28], p. 759). In part this flows from the characteristics of health organisations as ‘professional bureaucracies’ and separate ‘worlds’ described earlier [20]. As we saw, hospitals have traditionally been organised as ‘split hierarchies’ with doctors ‘remaining somewhat apart’ from administrative roles and responsibilities ([29], p. 560). The system of medical education and socialisation also tends to reinforce what Sinclair [30] described as an ‘exclusive professional identity’ linked to strong occupational cultures of ‘individualism’.

This cultural ambivalence towards management may be further exaggerated by a relative lack of incentives. The latter is especially true in those health systems where greater kudos is associated with research and where private income forms a large part of the total reward package of doctors, for instance, in Eastern Europe. Added to this are the limited career prospects for medical managers [4] not to mention the strains often associated with the job itself [15]. Indeed, it has been suggested that doctors who enter management roles frequently risk ‘professional isolation’ from colleagues, not to mention increased stress*.* Taking on a management role is thus a difficult psychological step, which moves the physician from a concern with their own performance to that of an institution they have no previous loyalty to.

This general absence of a strong ‘culture of engagement’ [31] towards management is reflected in numerous studies focusing on the way doctors respond to hybrid roles (such as clinical directors). However, as we noted earlier the literature also highlights are variable levels of enthusiasm and commitment towards management [32]. Most recently McGivern et al. [11] distinguish between ‘incidental hybrids’, oriented towards representing and protecting institutionalised professionalism and ‘willing hybrids’, who have more developed, stronger professional-management identities (also see the paper by Kuhlmann and colleagues in this volume].

These (sometimes radically) different reactions to management and leadership obviously beg the question of *how* we account for variation. In this area, the existing research is less well developed, although a number of themes are worth noting, including those relating to individual, organisational and wider institutional (including policy) levels of analysis. The former level suggests that the receptiveness of medical professionals may have much to do with individual characteristics such as age, specialist background and early career experiences [11]. More recently, research on the career backgrounds of medical managers in the English NHS finds that those reaching senior management positions (Medical Directors or CEOs) tend to be drawn almost exclusively from the ranks of the more prestigious (elite) specialisms (including surgery) and Universities [33].

A different kind of explanation has focused on the organisational context and the role of (non-clinical) general managers in fostering engagement. Bach ([34], p.110), for example describes how in three acute NHS trusts ‘chief executives tried to reinforce their authority by incorporating clinicians into the management process…’ the result being that ‘much of the hostility between managerial and medical staff had dissipated and a stronger sense of mutual interdependence… emerged’. These achievements, it is suggested, have much to do with the particular organisational cultures of individual hospitals, the policies adopted and the relative success of managers in drawing clinical professionals into the decision-making process. Indeed, it is suggested that organisational policies, shaped by general managers, may be key to influencing the *ability*, *motivation* and *opportunity* for doctors to engage with leadership roles [35].

Lastly it is important to note how the way doctors react to management imperatives may be linked to national level policies and institutions. It has been suggested for example, that low levels of medical commitment to management have been exaggerated by the way in which NPM reforms have been framed – as an assault on professions – and implemented in some contexts. Indeed, in the UK and elsewhere, there has been a strong sense of alienation arising from what many doctors believe to be the single-minded pursuit of financial targets. This fact may also have consequences for the way in which peak actors such as medical associations have responded to NPM reforms and the level of encouragement they have given for changes in the educational and training curriculum (a theme picked up in the paper by Hartley). In both respects, our attention is drawn to national level institutions that have been crucial in shaping both the nature of reforms and the way professionals respond to them. This topic is considered in some depth in the papers by Burau and by Denis and van Gestel.

### Outcomes and why?

A final area of concern relates to the outcomes of recent moves to increase the involvement of medicine in management. As we suggested earlier, this is assumed to have many benefits. Where governments and other payers are concerned it offers a possibility of enhanced control, essentially turning poachers into gamekeepers [36]. Co-opting doctors into management and leadership, it is argued, provides a low cost means of regulation, enlisting the support of doctors as ‘Chaser elites’ [37] who, are more able to influence practice and gain compliance amongst communities of fellow professionals that are ‘hard to reach’ [38]. This may ensure more systematic forms of (judgemental) supervision and responsible practice, through clinical audit and performance appraisal. It may also lead to stronger cost control, mainstreaming financial considerations into clinical judgements regarding diagnosis and treatment [13].

However, the assumed benefits of greater medical engagement in management and leadership go beyond these narrow objectives of control. As we have suggested already, it is also believed that this process will have marked consequences for the quality of services, both in terms of clinical outcomes and levels of patient satisfaction and wellbeing [4]. Hence, it is assumed that involving doctors in management will unleash their leadership potential with positive consequences for innovation. This might be in the form of service re-design around care pathways and improved teamwork with other (clinical) professions (such as nursing). In a similar way, the participation of doctors in management is assumed to have benefits for user *voice*. Here the argument is that clinical leaders will internalise the values of consumerism that have been central to the reforms, identifying more strongly with the goals of macro care of populations as opposed to the traditional clinical focus on the micro care of individuals. Clinical involvement in management might also strengthen ‘voice’ by helping to protect patients against managerial shortcuts that would otherwise endanger their safety and quality of care [39].

These outcomes of greater medical involvement in management and leadership are due, in part, it is argued, to the greater knowledge of the core business of hospitals, thus helping to develop service improvement plans which are better informed and targeted. The impact of board members with a clinical background may also be attributable to the enhanced credibility of clinical leaders helping to increase the likelihood that changes will be accepted and implemented by their colleagues. As Goodall ([40], p. 538) suggests ‘a doctor-leader who has spent years as a medical practitioner has acquired integrity that implies “walking the walk” which enhances a leader’s credibility.’ This, in turn, may have a symbolic effect, helping to fostering stronger professional engagement at lower levels, thus making it even more likely that service improvement initiatives will be implemented [4]. Indeed, it is suggested that hybrid professional managers may play a key ‘knowledge brokering role’ using their influence to communicate new innovations within and between organisations, helping to translate them into practice [41].

However, these outcomes should not necessarily be assumed. A potential downside of medical involvement in leadership is that it could simply reinforce older patterns of ‘custodial’ administration and defensive professionalism. This is especially if medical managers adopt strong advocacy roles, favouring their own speciality over and above wider ‘corporate’ interests. A risk here is that doctors seek to use their control over management work as ‘a stratagem for ensuring that no fundamental challenge is posed to their prevailing view of the world’ [29].

Hence, there are reasons to question the idea that deeper medical involvement in management and leadership activities will necessarily deliver intended results. Some research on this topic has drawn very positive results about the likely impact of doctors on hospital performance, especially at senior levels [40]. The paper by Rotar and colleagues draws very similar conclusions, focusing on hospitals in seven European health systems. However, as the paper by Sarto and Veronesi reveals, while there is a growing volume of research exploring the performance benefits of clinical leadership – much of it focused on board levels – not all studies point in the same direction.

### Summary of contributions

The papers that follow in this volume all, in different, ways contribute to the broad questions and concerns we have outlined so far. In total eight substantive papers are included, some reporting original research and others providing systematic overviews of the literature in given fields. In keeping with the objectives of the volume all the papers have a comparative focus, providing insights into the nature and impact of hospital management reforms and changing professional roles in a variety of national contexts. Specifically, the papers are concerned with four primary themes.

First, are wide ranging questions about the changing organisation and funding of health systems. This theme is taken up in the paper by Jeurissen, Duran and Saltman which explores a series of ‘uncomfortable realities’ faced by the European hospital sector, linked to changing payment regimes and demands for privatisation. The authors conclude that current policies may fall short in delivering better quality of care or lower costs and argue that policymakers will need to strike a balance between reforming the system and maintaining the capacity and ability of hospitals to deliver quality healthcare. As such, this paper helps to map out the organisational context in which management reforms are being implemented and how this in turn might have consequences for the changing roles and practices of clinical professionals.

Building on this, a second theme addressed in the volume is the question of how clinical leadership and management roles are developing internationally. The paper by Burau sets the scene for this discussion, by reviewing existing comparative research and assessing the strengths and weaknesses of different approaches. She concludes that a majority of studies exploring medicine and management adopt a macro level perspective, focusing on constraining institutions and the importance of path dependency in the way relationships between medicine and management unfold. However, Burau also identifies an emerging alternative meso level approach to comparative research which focuses more on processes of change and the agency of key actors (such as clinical professions) in shaping different outcomes. Future work, she suggests, needs to give more attention to this process model in order to break away from overly fixed, linear understandings of health management reform.

This focus on comparative dimensions of change is also central to the papers by Hartley and Denis and van Gestel. A starting point for Hartley is the growing focus across Europe (and more widely) on formal education and training for doctors in management and leadership. Following a review of the available literature on this topic and the results of an online survey of six country representatives involved in the European Association of Senior Hospital Physicians, she proposes a framework for comparing how management and leadership education is being approached within healthcare systems. By contrast, the paper by Denis and van Gestel is focused more specifically on comparing the challenges associated with engaging clinical professionals in two countries: Canada and the Netherlands. These cases were selected according to their level of institutional pluralism: one national health insurance system (Canada), and one etatist social insurance system (Netherlands). A key conclusion is that while the policy challenges are essentially the same, the method of implementation and response of medical professionals have been quite different. While in Canada there is evidence of an emerging trend towards more joint collaboration between governments and medical associations, this is less apparent in the Netherlands, where change has tended to be imposed top down.

The third theme explored by contributions to this volume relates to how medical professionals have reacted to management within organisational settings. This topic is addressed first in the paper by Kuhlmann, Rangnitt and von Knorring which looks at the under-researched issue of the organizational effects of attempts to co-opt doctors into management roles. Adopting a multi-level perspective (macro, meso and organisational) they investigate two case studies of management reform in Sweden. They conclude that while bringing doctors into management may hybridize formal roles, it does not necessarily change the perceptions of doctors themselves or improve managerial–professional coordination. In a similar vein the paper by Lega and Sartirana looks at changing relationships between medicine and management in the Italian NHS. Interestingly, the authors show how, in the Italian case efforts to engage frontline professionals in management spread, without deliberate planning. Greater medical engagement, they suggest, has been a consequence of doctors initiating new innovations in service provision in response to changes in the healthcare sector, thus making reform more consistent with professional logics.

Many of these concerns are also picked up in the paper by Correia and Denis, focusing on the implementation of clinical directorate structures within a public hospital in Portugal. The authors note how these new structures - which they depict as very similar to Minztberg’s ‘Multi-divisional’ form - have reinforced differences between doctors in management roles and rank and file colleagues. However, at the same time there are also signs that doctors may, to some extent, have captured these structures and are using them to bolster their traditional autonomy within hospitals.

The last main theme addressed by papers in this volume relates to the impact of changing levels of medical involvement in management on the performance of health services. This concern is taken up first in the paper by Sarto and Veronesi which provides a systematic review of the research evidence relating to hospital governance. This review focuses on scientific papers published in English in international journals and conference proceedings, extracted through a Boolean search strategy. Sarto and Veronesi conclude that in general terms, the findings show a positive impact of clinical leadership on different types of outcome measures, with only a handful of studies highlighting a negative impact on financial and social performance. Lastly, similar conclusions are drawn in the final paper by Rotar, Botje, Klazinga, Lombarts, Groene, Sunol and Plochg.^.^ Reporting on the results of a major European Union funded project (DUQue) the authors compare medical involvement in hospital governance in 19 OECD countries and then investigate the impact of this on quality management systems in a smaller sample of seven countries. The results indicate that where doctor managers have formal decision making responsibilities, this is positively associated with the level of implementation of quality management systems.

## Conclusions

The aim of this scene setting paper has been to raise a number of critical questions that might frame research on the changing relationship between medicine and management and which are addressed in many of the contributions that follow. Our conclusion is that while considerable work has been conducted on this topic, there remains scope for further investigation, especially in a comparative perspective. We also suggest that future work might benefit from a stringer engagement between different disciplines, most notably health policy, management and organisational theory. The potential for this synthesis is highlighted by many of the contributions to the special issue, as are the benefits of drawing on a wider range of data sources to chart both the development of medical manager roles and their impact.
